# Outcomes of Press-Fit Use of a Cementable Monoblock Radial Head Prosthesis in Complex Elbow Trauma: A Retrospective Observational Study

**DOI:** 10.7759/cureus.111919

**Published:** 2026-07-01

**Authors:** Rodrigo A Beraldo, Caroline Izidorio Bernardes Silva, Ana L Junho Lopes, Leonardo G Albano, Omar S Hafiz Hadaya, Ewerton A Galdeano, Daniel Giner Roselis, Renato Moraes

**Affiliations:** 1 Orthopedics and Traumatology, Instituto Jundiaiense de Ortopedia e Traumatologia, Jundiai, BRA; 2 Orthopedics and Traumatology, Hospital das Clínicas da Faculdade de Medicina da Universidade de São Paulo, Faculty of Medicine, University of São Paulo, São Paulo, BRA; 3 Nucleus of Education and Research, Hospital de Caridade São Vicente de Paulo, Jundiai, BRA

**Keywords:** complex elbow fractures, monoblock prosthesis, off-label use, periprosthetic radiolucency, press-fit fixation, radial head arthroplasty, terrible triad

## Abstract

Background: Radial head arthroplasty is the preferred surgical strategy for unreconstructable radial head fractures associated with complex elbow injuries. Single-diameter monoblock prostheses remain widely used and are sometimes implanted off-label as press-fit, even when the manufacturer's instructions specify cementation. Outcome data on this practice are scarce. We aimed to describe the clinical, functional, and radiographic outcomes of a single-diameter monoblock partial radial head prosthesis implanted off-label as a press-fit for complex elbow trauma.

Methods: We retrospectively reviewed 35 consecutive adult patients treated with the same single-diameter monoblock partial radial head prosthesis for complex elbow trauma between January 2021 and January 2024 at a tertiary referral center. The implant was used off-label, as the manufacturer's instructions specify bone-cement fixation. Injury patterns included terrible triad, Monteggia variants, transulnar fractures involving the coronoid base, and isolated irreparable radial head fractures. The primary outcomes were periprosthetic radiolucency and implant loosening at the 24-month radiograph and the association between them; secondary outcomes were the Mayo Elbow Performance Score (MEPS), range of motion, visual analog scale (VAS) for pain, return to work, and postoperative complications. The association between radiolucency and loosening was tested with Fisher's exact test (two-tailed).

Results: The mean age was 48.5 ± 13.6 years (range, 27-75); 20 (57.1%) were male patients, and the dominant limb was injured in 13 patients (37.1%). The most frequent injury pattern was the terrible triad (60.0%). With a minimum follow-up of two years and a mean follow-up of 30 months (range, 24-36 months), mean elbow flexion was 117.7°± 13.9°, mean extension deficit was 20.3°± 17.0°, and mean combined forearm rotation was 138.9°. A functional arc of motion was achieved in 57.1%. MEPS was good or excellent in 22 patients (62.9%; 95% CI, 46.3-76.8); mean VAS pain at last follow-up was 2.0 ± 1.7 (range, 0-6); 25 (71.4%) returned to their previous occupation. Periprosthetic radiolucency was identified in 30 (85.7%) and implant loosening in 15 (42.9%; 95% CI, 27.7-59.4%); interrater agreement between two blinded elbow specialists who did not perform any of the surgeries was almost perfect (Cohen’s κ = 0.91 (95% CI, 0.81-1.00) for radiolucency and κ = 0.93 (95% CI, 0.85-1.00) for loosening). All patients with loosening exhibited radiolucency; however, this association did not reach statistical significance on a two-tailed Fisher’s exact test (p = 0.057). Postoperative deep infection occurred in one patient (2.9%); no permanent neurological injury was recorded; four patients (11.4%) underwent reoperation (one infection, one stiffness, one instability, one symptomatic complication managed with hardware removal).

Conclusion: Off-label press-fit use of a cementable monoblock radial head prosthesis produced satisfactory short- to mid-term functional outcomes with an acceptable complication profile. Although periprosthetic radiolucency was highly prevalent and implant loosening occurred in nearly half the cohort, most radiographic findings remained clinically silent, and reoperation was infrequent. These data support the press-fit use of this implant as a viable option in resource-limited settings, while highlighting the importance of systematic radiographic surveillance and longer-term follow-up.

## Introduction

Radial head fractures account for approximately one-third of adult elbow fractures and represent a nonnegligible burden on hand and elbow surgical practice [1,2]. The radial head is a critical secondary stabilizer against valgus stress, contributes to axial load transmission, and is essential to forearm rotation [3]. Injury to the radial head, particularly when associated with collateral ligament disruption, coronoid fracture, or ulnohumeral dislocation, may result in residual instability, stiffness, and post-traumatic arthritis when not properly managed [4,5].

Treatment selection depends on fracture morphology, ligamentous integrity, and patient factors. Open reduction and internal fixation (ORIF) is appropriate when stable anatomic reconstruction is achievable, while isolated radial head excision in the setting of complex injuries has been associated with persistent instability and inferior functional outcomes [6,7]. In comminuted, unreconstructable fractures and unstable fracture-dislocation patterns, radial head arthroplasty (RHA) provides immediate mechanical stability, restores axial load distribution, and permits early mobilization [8,9].

Available radial head implants vary in design (monopolar vs. bipolar; monoblock vs. modular) and in fixation strategy (cemented vs. press-fit). Modular systems allow independent selection of head and stem dimensions, whereas monoblock designs provide structural rigidity and eliminate the risk of component dissociation. Fixation strategy may influence long-term radiographic behavior, and uncemented press-fit configurations have been associated with higher rates of periprosthetic radiolucency than cemented designs [10].

Monoblock implants designed for cemented fixation are sometimes implanted off-label as press-fit, particularly when surgeons prefer to avoid bone cement to facilitate eventual revision, or when intraoperative canal fit is judged adequate for primary stability. Outcomes of this off-label practice are scarcely documented [11]. The aim of this study was to describe the clinical, functional, and radiographic outcomes of a single-diameter monoblock partial radial head prosthesis implanted off-label as a press-fit for complex elbow fractures. The primary objectives were to determine the rates of periprosthetic radiolucency and implant loosening at the 24-month radiographic assessment and to test the association between them; secondary objectives were to characterize functional outcomes (Mayo Elbow Performance Score (MEPS)), range of motion, pain, return to work, and postoperative complications. We hypothesized that this practice would yield satisfactory short- to mid-term function despite a high incidence of radiographic changes and that radiolucency would be associated with implant loosening.

## Materials and methods

Study design

We conducted a retrospective observational cohort study reported in accordance with the Strengthening the Reporting of Observational Studies in Epidemiology statement [12]. Electronic medical records, radiographs, and standardized functional assessments were reviewed for all consecutive adult patients who underwent press-fit implantation of a single-diameter monoblock partial radial head prosthesis for complex elbow trauma between January 2021 and January 2024.

Setting

All procedures were performed at a tertiary referral center for trauma. All 35 surgeries were performed by two elbow surgeons, each with more than five years of dedicated experience in elbow surgery.

Ethics

The study was approved by the Research Ethics Committee of the Faculdade de Medicina de Jundiaí (Plataforma Brasil CAAE 88614625.4.0000.5412; approval no. 7.765.164, dated August 14, 2025) and conducted in accordance with the principles of the Declaration of Helsinki and Brazilian Resolution CNS 466/2012. Written informed consent was obtained from all participants prior to in-person functional and radiographic reevaluation.

Participants

Inclusion Criteria

Adults (≥18 years) treated with a single-diameter, monoblock partial radial head prosthesis as definitive management of a complex elbow fracture, defined as one of the following patterns, were included: terrible triad of the elbow [13], Monteggia variant; transulnar fracture-dislocation involving the coronoid base; or isolated irreparable radial head fracture (Mason-Hotchkiss type III-IV) [5]. A minimum follow-up of two years and complete clinical, functional, and radiographic data were required.

Exclusion Criteria

Follow-up shorter than two years, incomplete records, prior elbow surgery on the affected side, active infection, preexisting neurological deficit, or additional ipsilateral upper extremity injuries were the exclusion criteria.

Objectives

The primary objectives were 1) to determine the rates of periprosthetic radiolucency and implant loosening at the 24-month radiographic assessment and 2) to test the association between radiolucency and loosening. The secondary objectives were to characterize the categorical MEPS, active range of motion, the proportion of patients achieving a functional arc of motion, pain on a visual analog scale (VAS, 0-10), return to the previous occupation, and postoperative complications, including infection, neurological injury, and reoperation.

Implant

All patients received the same single-diameter, monoblock partial radial head prosthesis (Sartori; manufactured by Cortical Produtos para Cirurgia Ortopédica, São Carlos, São Paulo, Brazil). The implant is a one-piece metallic device featuring a beveled distal stem to facilitate insertion into the medullary canal and a highly polished articular head. According to the manufacturer's instructions for use, the implant is intended to be fixed to the radial medullary canal with bone cement. In the present series, the prosthesis was implanted off-label as a press-fit in all 35 patients, without bone cement, based on intraoperative assessment of adequate canal fit and the surgical team's preference for a cement-free interface to facilitate eventual revision.

Surgical technique

All procedures were performed under general anesthesia with the patient supine. A lateral Kocher approach was used in all cases, between the anconeus and the extensor carpi ulnaris. The radial head fragments were removed and measured, and the prosthetic head was selected to match the anatomic head dimensions, with care to avoid overstuffing and to align the prosthetic articular surface with the lesser sigmoid notch under direct vision and intraoperative fluoroscopy. The lateral collateral ligament complex was repaired to its humeral footprint with transosseous sutures or suture anchors when disrupted; coronoid fragments were addressed by suture lasso or fragment-specific fixation when amenable; and the annular ligament was repaired when disrupted. Stability was tested intraoperatively under fluoroscopy with valgus and varus stress and a lateral pivot shift maneuver. Wounds were closed in layers, and a posterior splint was applied.

Postoperative protocol

The posterior splint was maintained for 7-10 days, after which active and active-assisted range of motion was initiated under the supervision of an occupational therapist. Heavy lifting and contact sports were restricted for 12 weeks. Patients were followed clinically and radiographically at two and six weeks; three, six, twelve, and twenty-four months postoperatively; and yearly thereafter.

Variables

Demographic data included age, sex, side of injury, dominance of the affected limb, smoking status, and diabetes mellitus. Clinical and functional outcomes included active flexion, extension deficit, pronation, and supination measured with a goniometer at the final follow-up; the presence of a functional arc of motion (defined as 30°-130° flexion-extension and ≥50° pronation/supination); the categorical MEPS, as originally described by Morrey and An [14]; pain on a VAS (0-10); the presence of pain at rest and pain with occupational tasks; and return to the previous occupation.

Radiographic variables included the presence of periprosthetic radiolucency and implant loosening, assessed on standardized anteroposterior and lateral elbow radiographs obtained at two and six weeks; at three, six, twelve, and twenty-four months postoperatively; and yearly thereafter. The 24-month radiograph served as the index image for the radiographic outcome analysis. Periprosthetic radiolucency was defined as a radiolucent line ≥1 mm wide adjacent to the stem in any zone on either projection, persisting on at least two consecutive radiographs, criteria adapted from Popovic et al. [15]. Implant loosening was defined as progressive radiolucency >2 mm and/or stem migration, angular change of the stem axis, or subsidence on serial imaging. All radiographs were independently and blindly read by two shoulder and elbow surgeons with five and 15 years of dedicated experience, respectively, neither of whom had performed any of the index surgeries; disagreements were resolved by consensus, and interrater agreement was quantified with Cohen's κ statistic.

Statistical analysis

The normality of continuous variables was assessed with the Shapiro-Wilk test; all variables met the normality assumption and are reported as mean ± standard deviation with range. Categorical variables are reported as absolute frequencies and percentages, with 95% confidence intervals (CIs; Wilson method) for primary outcome proportions [16]. The association between periprosthetic radiolucency and implant loosening was tested with a two-tailed Fisher's exact test; the absolute risk difference was used as a measure of effect size. Interrater agreement for the radiographic variables was quantified with Cohen's κ statistic and interpreted according to the criteria of Landis and Koch [17], in which κ values are classified as poor (<0.00), slight (0.00-0.20), fair (0.21-0.40), moderate (0.41-0.60), substantial (0.61-0.80), and almost perfect (0.81-1.00). A two-tailed p value of <0.05 was considered statistically significant. Analyses were performed in IBM Statistical Package for the Social Sciences Statistics, version 20.0 (IBM Corp., Armonk, NY).

## Results

Baseline characteristics

**Figure 1:**
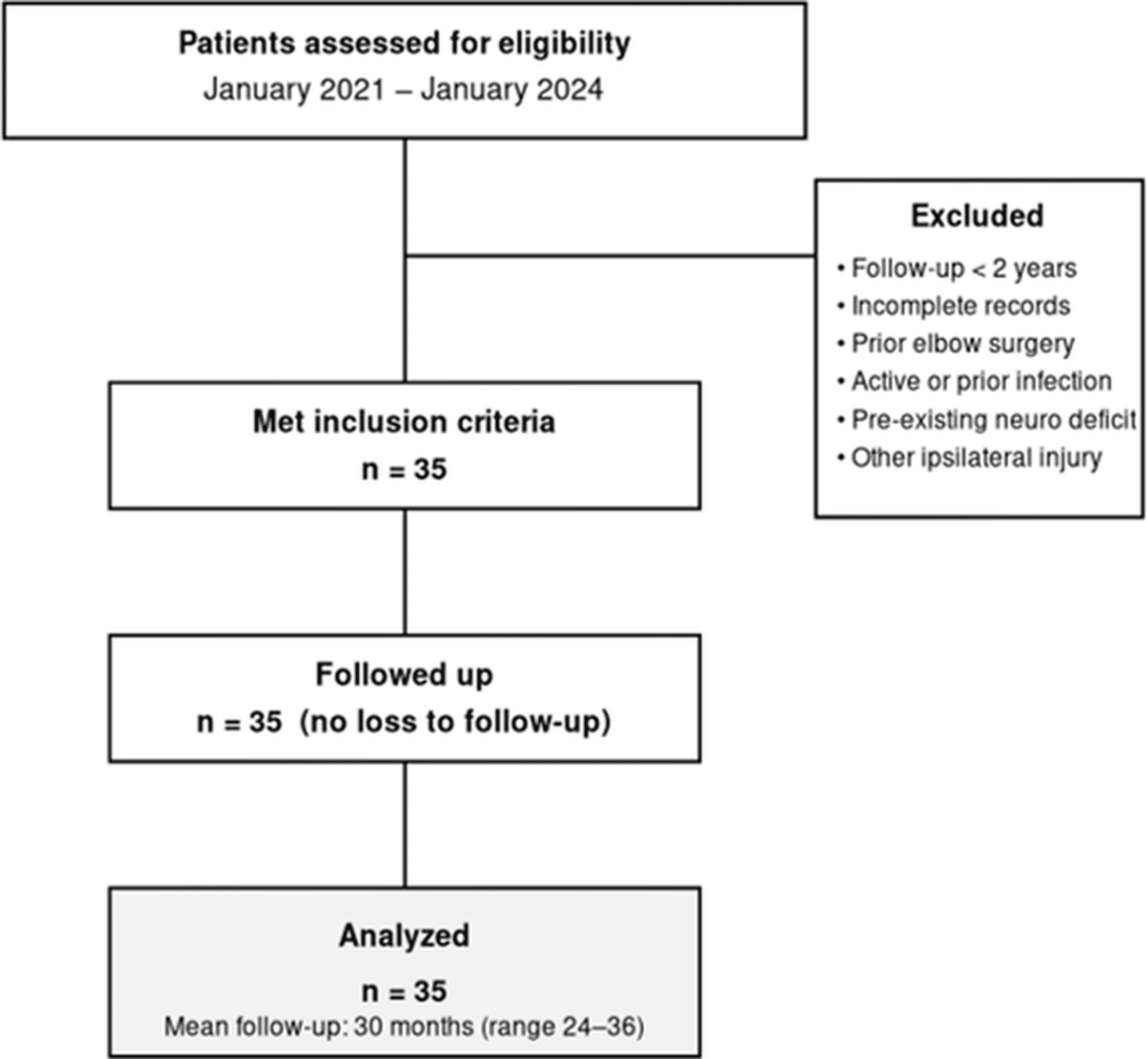
STROBE flow diagram of the study cohort Flow of participants through the study, presented in accordance with the STROBE statement for observational studies. Adult patients undergoing radial head arthroplasty with the Sartori monoblock partial radial head prosthesis between January 2021 and January 2024 were assessed for eligibility. After applying the inclusion and exclusion criteria, 35 consecutive patients were included, completed clinical and radiographic follow-up of at least two years, and were analyzed STROBE: Strengthening the Reporting of Observational Studies in Epidemiology

Thirty-five consecutive patients met the inclusion criteria and were analyzed (Figure 1). No patients were excluded for protocol deviation during the study period. The mean follow-up was 30 months (range, 24-36 months), with a minimum follow-up of two years for all included patients.

Mean age at surgery was 48.5 ± 13.6 years (range, 27-75); 20 (57.1%) were male patients. The injured side was the right in 15 patients (42.9%) and the left in 20 (57.1%). The dominant limb was injured in 13 patients (37.1%). One patient (2.9%) was an active smoker, and four (11.4%) had diabetes mellitus (Table 1). The most frequent injury pattern was the terrible triad of the elbow (21 patients, 60.0%), followed by Monteggia variants (6, 17.1%), transulnar fracture dislocations involving the coronoid base (4, 11.4%), and isolated irreparable radial head fractures (4, 11.4%) (Table 2).

**Table 1:** Demographic and clinical characteristics (n = 35) SD: standard deviation

Variable	Result
Age (years), mean ± SD (range)	48.5 ± 13.6 (27-75)
Gender, n (%)	
Male	20 (57.1%)
Female	15 (42.9%)
Right side injured, n (%)	15 (42.9%)
Left side injured, n (%)	20 (57.1%)
Dominant limb injured, n (%)	13 (37.1%)
Active smoker, n (%)	1 (2.9%)
Diabetes mellitus, n (%)	4 (11.4%)

**Table 2:** Distribution of injury patterns (n = 35)

Injury pattern	n (%)
Terrible triad of the elbow	21 (60.0%)
Monteggia variant	6 (17.1%)
Transulnar fracture-dislocation involving the coronoid base	4 (11.4%)
Isolated irreparable radial head fracture (Mason-Hotchkiss III-IV)	4 (11.4%)

Primary outcomes

Radiographic Findings

At the 24-month radiographic assessment, periprosthetic radiolucency was identified in 30 patients (85.7%; 95% CI, 70.6-93.7) and implant loosening in 15 (42.9%; 95% CI, 27.7-59.4) (Table 3). Interrater agreement between the two blinded elbow surgeons was almost perfect, with Cohen’s κ = 0.91 (95% CI, 0.81-1.00) for radiolucency and κ = 0.93 (95% CI, 0.85-1.00) for loosening. All patients with loosening also exhibited radiolucency, while none of the five patients without radiolucency developed loosening; however, this difference did not reach statistical significance on a two-tailed Fisher’s exact test (p = 0.057; Table 4). As a measure of effect size, the absolute risk difference in loosening between patients with and without radiolucency was 50 percentage points (15/30 (50%) vs. 0/5 (0%)); the small subgroup of patients without radiolucency (n = 5) limits the precision of this estimate. The negative predictive value of radiolucency absence for the absence of loosening was 100% (5/5).

**Table 3:** Pain, return to work, radiographic findings, and complications (n = 35) CI: confidence interval (Wilson method); SD: standard deviation; VAS: visual analog scale

Variable	Result
VAS pain at final follow-up, mean ± SD (range) (95% CI)	2.0 ± 1.7 (0-6)
Pain at rest, n (%)	7 (20.0%)
Pain with occupational tasks, n (%)	27 (77.1%)
Returned to previous occupation, n (%) (95% CI)	25 (71.4%) (55.0-83.5)
Periprosthetic radiolucency, n (%) (95% CI)	30 (85.7%) (70.6-93.7)
Implant loosening, n (%) (95% CI)	15 (42.9%) (27.7-59.4)
Postoperative deep infection, n (%)	1 (2.9%)
Permanent neurological injury, n (%)	0 (0.0%)
Reoperation, n (%)	4 (11.4%)

**Table 4:** Association between periprosthetic radiolucency and implant loosening (n = 35) Fisher's exact test: two-tailed p = 0.057

Periprosthetic radiolucency	No loosening	Loosening	Total
Absent	5	0	5
Present	15	15	30
Total	20	15	35

Secondary outcomes

Mayo Elbow Performance Score

Categorical MEPS was distributed as follows: excellent in nine patients (25.7%), good in 13 (37.1%), fair in 10 (28.6%), and poor in three (8.6%). The combined good-or-excellent proportion was 62.9% (22/35; 95% CI, 46.3-76.8%) (Table 5).

**Table 5:** Categorical MEPS at final follow-up (n = 35) CI: confidence interval (Wilson method); MEPS: Mayo Elbow Performance Score

MEPS category	Value, n (%)
Excellent (90-100)	9 (25.7%)
Good (75-89)	13 (37.1%)
Fair (60-74)	10 (28.6%)
Poor (<60)	3 (8.6%)
Good or excellent (combined)	22 (62.9%; 95% CI, 46.3-76.8)

Range of Motion and Functional Arc

At final follow-up, mean active elbow flexion was 117.7° ± 13.9° (range, 80°-140°), with a mean extension deficit of 20.3° ± 17.0° (range, 0°-70°). Mean active forearm pronation and supination were 70.6° ± 12.1° and 68.3° ± 18.2°, respectively, yielding a mean combined rotational arc of 138.9°. A functional arc of motion was achieved by 20 patients (57.1%) (Table 6).

**Table 6:** Range of motion at final follow-up (n = 35) ^*^Functional arc defined as 30°-130° flexion-extension and ≥50° pronation/supination SD: standard deviation

Variable	Value, mean ± SD (range)
Active flexion (°)	117.7 ± 13.9 (80-140)
Extension deficit (°)	20.3 ± 17.0 (0-70)
Pronation (°)	70.6 ± 12.1 (10-80)
Supination (°)	68.3 ± 18.2 (0-85)
Functional arc achieved, n (%)^*^	20 (57.1%)

Pain and Return to Work

Mean VAS pain at final follow-up was 2.0 ± 1.7 (range, 0-6). Pain at rest was reported by seven patients (20.0%) and pain with occupational tasks by 27 patients (77.1%). Twenty-five patients (71.4%; 95% CI, 55.0-83.5) returned to their previous occupation.

Complications and reoperation

One patient (2.9%) developed a postoperative deep infection. No patient sustained a permanent neurological injury. Four patients (11.4%) underwent reoperation: one for deep infection (debridement and implant removal), one for stiffness (open arthrolysis), one for late instability (revision surgery), and one for symptomatic complications managed with hardware removal. Indications and details of each reoperated case are summarized in Table 7.

**Table 7:** Reoperated cases (n = 4) Cases are listed in chronological order of reoperation F: female; M: male

Case	Age (years)/sex	Injury	Radiolucency/loosening	Indication	Procedure/date
1	43/M	Terrible triad	Yes/Yes	Deep infection	Debridement + implant removal (Mid July 2022)
2	27/M	Terrible triad	Yes/Yes	Stiffness	Open arthrolysis (Mid March 2024)
3	45/F	Terrible triad	Yes/Yes	Late instability	Revision surgery (End July 2023)
4	32/M	Monteggia variant	Yes/No	Symptomatic - hardware removal	Hardware removal, right elbow (Mid September 2024)

## Discussion

The principal finding of this consecutive series of 35 patients with complex elbow trauma is that off-label press-fit use of a cementable monoblock partial radial head prosthesis produced a high prevalence of periprosthetic radiolucency and a moderate rate of implant loosening on radiographic follow-up, in the context of satisfactory short- to mid-term functional outcomes and an acceptable complication profile. Most radiographic changes were clinically silent: only four patients (11.4%) underwent reoperation, and the dissociation between radiographic and clinical outcomes is one of the most clinically relevant observations of this work.

With respect to the primary radiographic outcomes, periprosthetic radiolucency (85.7%) and implant loosening (42.9%) were both at the upper end of the published range for fixed-stem prostheses. Vannabouathong et al., in a systematic review and meta-analysis, demonstrated that uncemented and cemented designs are not equivalent and that periprosthetic stress shielding and lucent lines are common with press-fit configurations [10]. Samra et al. reported a high mid-term loosening rate with anatomic monopolar press-fit prostheses, prompting caution regarding the routine use of this fixation strategy [18]. Ha et al. previously documented radiographic changes in approximately 88% of RHA cases on long-term follow-up, although clinical correlation was inconsistent [19]. Popovic et al. described progressive radiolucent line formation at the bone-cement interface of bipolar prostheses, with some clinically apparent osteolysis at midterm [15]. Our findings align with this body of evidence: radiolucency and loosening are common with single-diameter monoblock designs implanted as press-fit. Although the association between radiolucency and loosening did not reach statistical significance on a two-tailed Fisher's exact test (p = 0.057) and should therefore be interpreted with caution, the biological plausibility of this relationship is supported by the fact that all patients with loosening exhibited radiolucency. As an effect size measure, the absolute risk difference in loosening between patients with and without radiolucency was 50 percentage points (15/30 (50%) vs. 0/5 (0%)); however, the small subgroup without radiolucency (n = 5) limits the precision of this estimate. The 100% negative predictive value of an absence of radiolucency for an absence of loosening, while based on a small subgroup of five patients, supports the role of routine radiographic surveillance as a screening tool.

Several factors specific to the present series may explain the elevated radiographic findings. First, the implant was used off-label: the manufacturer intended it for cemented fixation, but the surgical team chose press-fit implantation in all 35 cases. Although press-fit fixation simplifies implantation and avoids the long-term concerns associated with cement debris and revision difficulty, primary stability depends critically on accurate intraoperative sizing and on the quality of the proximal radial cortical bone. The interface between a polished, beveled stem and the relatively short and narrow radial canal may offer less rotational stability than purpose-designed press-fit stems with porous coatings or hydroxyapatite, increasing the likelihood of micromotion and lucent-line development. Second, monoblock geometry precludes independent adjustment of head and stem dimensions, which may compromise canal fill and radiocapitellar congruence [10]. Modular monopolar designs, which permit independent selection of head and stem, have generally reported lower loosening rates (5%-30% across series) [9,20]. Bipolar designs raise different concerns: although they may accommodate radiocapitellar mismatch, they have been associated with osteolysis and bone-cement interface failure when cemented [15,21] and remain debated in the management of fracture-dislocations [22]. Despite these design and technique-related considerations, the dissociation between radiographic loosening and clinical symptoms, reflected in the low reoperation rate, suggests that radiographic loosening alone, in the absence of pain, instability, or functional deterioration, should not be considered a sufficient indication for revision surgery. This conclusion echoes that of Lobo-Escolar et al. [23] and is consistent with the broader literature on asymptomatic periprosthetic changes.

Among the secondary outcomes, functional results in this cohort are broadly comparable to those reported in the contemporary RHA literature. Heijink et al., in a systematic review of 38 studies, described mean flexion-extension arcs of approximately 100°-130° and rotational arcs of 120° or greater across implant designs, with good or excellent MEPS in the majority of patients [9]. Catellani et al., in a systematic review of Mason III-IV fractures, demonstrated superior outcomes with arthroplasty over isolated resection [7]. In the press-fit subset, Lobo-Escolar et al. reported satisfactory outcomes in 21 complex radial head fractures treated with a press-fit prosthesis [23]. Le Mapihan et al., in a larger cohort of 56 patients managed with a short-cemented bipolar implant, achieved a mean MEPS of 88 and a flexion-extension arc of 117° at minimum two-year follow-up [24]; Celli et al., in long-term follow-up (mean 149 months) of 23 bipolar Judet prostheses, reported a mean MEPS of 88 with a 26% revision rate [25]. Our combined arc of 117.7° flexion with a 20.3° extension deficit and 138.9° rotational arc, together with 62.9% good-or-excellent MEPS, mean VAS of 2.0, and 71.4% return to work, places the present cohort in the same range, despite a fracture pattern distribution dominated by complex fracture-dislocations (terrible triad and Monteggia variants accounted for 77.1% of cases).

The complication profile of this cohort was consistent with reported standards for RHA in similar populations. The infection rate of 2.9% and the absence of permanent neurological injury are concordant with rates in published cohorts [9,24]. Reoperation in 11.4% is within the 5%-20% range typically reported for RHA in complex elbow trauma [9,25], and the indications observed in the present series (infection, stiffness, instability, symptomatic loosening) reflect the spectrum described by Eyberg and McKee [11].

From a clinical and health-system perspective, our findings are particularly relevant to healthcare environments where supply, reimbursement, and inventory constraints frequently dictate implant availability. Off-label press-fit use of a cementable monoblock prosthesis appears to deliver functional outcomes comparable to those of dedicated press-fit and cemented designs, at substantially lower cost. Surgeons electing this strategy should counsel patients on the high probability of radiographic changes, implement systematic radiographic follow-up, and reserve revision surgery for symptomatic cases. Future studies should compare press-fit and cemented use of the same monoblock implant in a controlled fashion to define the impact of fixation choice on long-term implant survival.

Limitations

This study has several limitations. First, its retrospective design introduces selection and information bias. Second, the sample size of 35 patients limits statistical power for subgroup comparisons and multivariable modeling. Third, the mean follow-up of 30 months (range 24-36 months) is sufficient to capture mid-term complications but precludes definitive conclusions on long-term implant survival. Fourth, MEPS was reported in categorical form rather than continuously, reducing the granularity of functional comparisons. Fifth, the absence of a comparison group (cemented use of the same implant, modular RHA, bipolar RHA, or ORIF) limits direct inference about the relative merits of off-label press-fit fixation. Finally, the study reflects practice at a single tertiary center and may not generalize to all surgical environments.

Strengths

Strengths include the homogeneity of the cohort: all 35 surgeries were performed by two elbow surgeons with more than five years of dedicated experience, using a single implant, a single fixation strategy, and a single rehabilitation protocol. Radiographic outcomes were assessed independently and blindly by two additional shoulder and elbow surgeons with five and 15 years of experience, neither of whom had performed any of the index surgeries, achieving almost perfect interrater agreement (Cohen's κ = 0.91 (95% CI, 0.81-1.00) for radiolucency and κ = 0.93 (95% CI, 0.85-1.00) for loosening) and mitigating the interpretive bias frequently associated with single-reader designs. The study systematically evaluated the radiolucency-loosening relationship, and the fracture pattern distribution is dominated by complex fracture-dislocations, reflecting real-world referral practice in elbow trauma. To our knowledge, this is the first published series specifically describing the off-label press-fit use of a cementable monoblock radial head prosthesis.

## Conclusions

In this consecutive series of 35 patients with complex elbow trauma, off-label press-fit implantation of a monoblock partial radial head prosthesis designed for cemented fixation was associated with a high prevalence of periprosthetic radiolucency (85.7%; 95% CI, 70.6-93.7) and a moderate rate of implant loosening (42.9%; 95% CI, 27.7-59.4) at the 24-month radiographic assessment. The association between radiolucency and loosening did not reach statistical significance on a two-tailed Fisher's exact test (p = 0.057), and this result should be interpreted with caution. Most radiographic findings were clinically silent, and the cohort achieved satisfactory functional outcomes (62.9% good or excellent on MEPS; mean VAS pain 2.0; 71.4% return to work) with a low reoperation rate (11.4%) at short- to mid-term follow-up.

These findings have several clinical implications. First, surgeons considering this off-label press-fit technique should counsel patients about the high probability of radiographic changes and implement systematic radiographic surveillance, reserving revision surgery for cases with progressive pain, instability, or functional deterioration, not for radiographic loosening alone. Second, despite elevated rates of radiographic change, functional outcomes remained comparable to those reported for dedicated press-fit and cemented RHA designs, suggesting that this approach is a viable option in resource-limited or cost-constrained settings where dedicated implants may not be available. Third, the near-perfect interrater agreement (Cohen's κ ≥ 0.91) supports the reliability of the radiographic criteria used. Future studies should compare press-fit and cemented use of the same monoblock implant in a controlled design, and longer follow-up is needed to determine whether these radiographic changes progress and ultimately affect functional outcomes or implant survival.
